# Weight compensation characteristics of Armeo®Spring exoskeleton: implications for clinical practice and research

**DOI:** 10.1186/s12984-017-0227-0

**Published:** 2017-02-17

**Authors:** Bonnie E. Perry, Emily K. Evans, Dobrivoje S. Stokic

**Affiliations:** Center for Neuroscience and Neurological Recovery, Methodist Rehabilitation Center, 1350 East Woodrow Wilson Drive, Jackson, MS 39216 USA

**Keywords:** Upper extremity, Exoskeleton, Robotics, Weight compensation, Neurorehabilitation

## Abstract

**Background:**

Armeo®Spring exoskeleton is widely used for upper extremity rehabilitation; however, weight compensation provided by the device appears insufficiently characterized to fully utilize it in clinical and research settings.

**Methods:**

Weight compensation was quantified by measuring static force in the sagittal plane with a load cell attached to the elbow joint of Armeo®Spring. All upper spring settings were examined in 5° increments at the minimum, maximum, and two intermediate upper and lower module length settings, while keeping the lower spring at minimum. The same measurements were made for minimum upper spring setting and maximum lower spring setting at minimum and maximum module lengths. Weight compensation was plotted against upper module angles, and slope was analyzed for each condition.

**Results:**

The Armeo®Spring design prompted defining the slack angle and exoskeleton balance angle, which, depending on spring and length settings, divide the operating range into different unloading and loading regions. Higher spring tensions and shorter module lengths provided greater unloading (≤6.32 kg of support). Weight compensation slope decreased faster with shorter length settings (minimum length = −0.082 ± 0.002 kg/°; maximum length = −0.046 ± 0.001 kg/°) independent of spring settings.

**Conclusions:**

Understanding the impact of different settings on the Armeo®Spring weight compensation should help define best clinical practice and improve fidelity of research.

**Electronic supplementary material:**

The online version of this article (doi:10.1186/s12984-017-0227-0) contains supplementary material, which is available to authorized users.

## Background

Robotic technology has been increasingly used for assessing and treating upper extremity motor deficits after a neurological injury. Robotic therapy offers high intensity training [1, 2], one of the key determinants of motor recovery. When combined with conventional therapy [2], robotic therapy yields largely favorable outcomes in terms of improving motor control [1–4], reducing motor deficits, and increasing ability to carry out activities of daily living [5, 6].

Robotic devices can be classified as end-effectors or exoskeletons, interfacing with the distal joint only or aligned with both proximal and distal joints, respectively [3, 5]. They are generally described as providing assistance to complete a desired movement or resistance to dampen or prevent undesired movements or deviations from a predetermined path [7]. Assistive therapy in the form of weight compensation has been implemented using powered or spring-based exoskeletons. Increasing the amount of weight compensation has been shown to increase range of motion [8] and decrease muscle activation [9–11], which has been associated with decoupling flexor synergies after stroke [12].

The Armeo®Spring is a widely used arm rehabilitation device with approximately 800 adult and pediatric units installed worldwide (personal communication with Hocoma, Inc). It is a spring-based weight compensation exoskeleton that allows virtual gaming in a three-dimensional workspace. The exoskeleton consists of an upper module for the upper arm, a lower module for the forearm, and a pressure sensitive handgrip. Each module is length adjustable to align the exoskeleton with the arm joints and is equipped with a spring that provides adjustable weight compensation across nine settings (A-I).

To our knowledge, in depth information on the amount of weight compensation provided by different spring settings is not readily available, either in the Armeo®Spring manual or in the literature. In the study investigating the efficacy of the T-Wrex (Therapy Wilmington Research Exoskeleton), the Armeo®Spring research predecessor, the investigators standardized initial position of the arm and progressively reduced weight compensation by 40% over 8 weeks [13]. However, absolute weight compensation was not stated. Three other studies also standardized the starting arm position, but the spring settings to achieve that were not reported [14–16]. Another study reported using 40% weight compensation throughout the study, without detailing how it was determined [17]. None of the studies reported module length settings used across participants. Such incomplete reporting is understandable since precise information on weight compensation provided by different spring tension and module length settings is not readily available. Without this information, however, it is difficult for clinicians and researchers to develop standardized treatment protocols, appreciate outcomes, and replicate studies.

The purpose of this study was to assess weight compensation throughout the operating range of the Armeo®Spring exoskeleton by systematically changing spring tension and module length settings. Our main focus was on the effect of the upper module spring because of the specific design and greater need for compensating the upper arm weight. Based on the laws of mechanics, we hypothesized that the amount of weight compensation would increase with increasing spring tension and decreasing exoskeleton length. The implications of findings are discussed from clinical and research perspectives. This knowledge is expected to provide a framework for developing better treatment plans and research protocols for the Armeo®Spring.

## Methods

### Setup and instrumentation

**Fig. 1:**
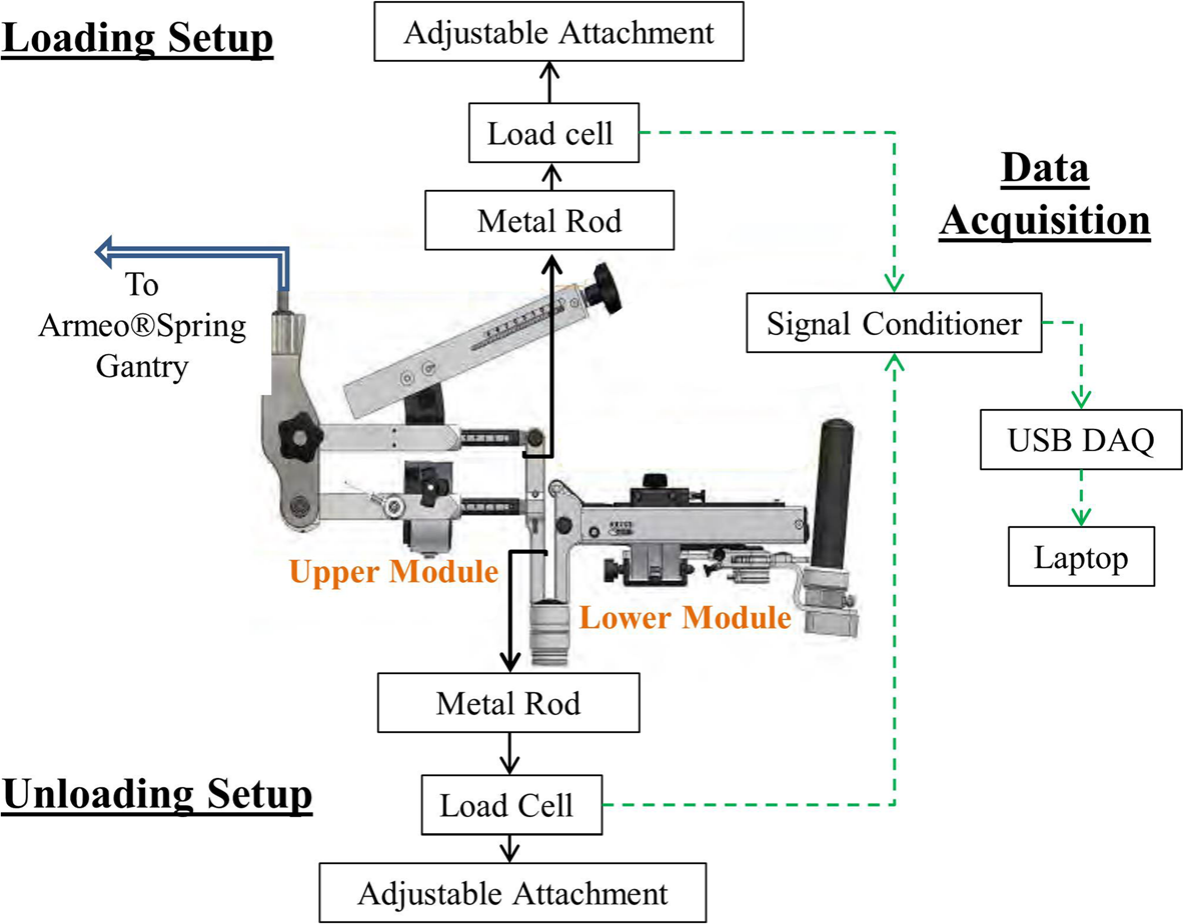
Experimental Setup

The net static force applied to a load cell was measured to infer the weight compensation provided by the springs at different settings in the sagittal plane (Fig. 1). Both shoulder and elbow joints of the exoskeleton were locked to maintain the upper and lower modules in the horizontal plane. The load cell was connected near the elbow joint and kept vertical to the exoskeleton. To accommodate the direction of spring forces exerted on the load cell, the instrumentation was positioned below the exoskeleton to measure unloading and above the exoskeleton to measure loading.

The load cell (Omega LCCB200) was connected to a signal conditioner (Daytronic 3270). The output voltage was fed into an analog-to-digital converter (National Instruments USB-6008) and recorded in a custom LabView program for 10 s per condition (sample rate 1 kHz). The load cell was calibrated to derive a linear formula for converting voltage into weight.

To ensure generalizability of results, the spring constant was compared to the constant of a new replacement spring using Hook’s law. The two constants were not significantly different (mean ± standard error: exoskeleton spring k = 3935 ± 140.6 N/m, replacement spring k = 3768 ± 75.08 N/m, *p* = 0.31).

### Protocol

Weight compensation provided by the Armeo®Spring adult version (v2.0) was measured for all nine upper spring settings (A-I) at four length settings of the upper (U) and lower (L) exoskeleton modules, with the lower spring held at the minimum (A). The length settings were specified by the numbers marked on each module (upper = 1-10; lower = 1–12), referring to increments in centimeters. The minimum, maximum, and 2 intermediate length settings were selected for each module, denoted here as U1L1, U10L12, U4L5, and U7L8, respectively. Minimum and maximum length settings were chosen to characterize the weight compensation at the extremes of the device, while the intermediary lengths U4L5 and U7L8 were selected as proportional increments between the two extremes to cover the full range of the device. To examine the greatest impact of the lower module spring, the maximum setting (E) was selected, and data were collected at lengths U1L1 and U10L12, while keeping the upper spring at the minimum (A). For each condition, data were collected at 5° increments selected randomly throughout the upper module operating range (−40° to +40° relative to horizontal). The Armeo®Spring sensor which monitors shoulder flexion was used to measure upper module angle.

**Fig. 2:**
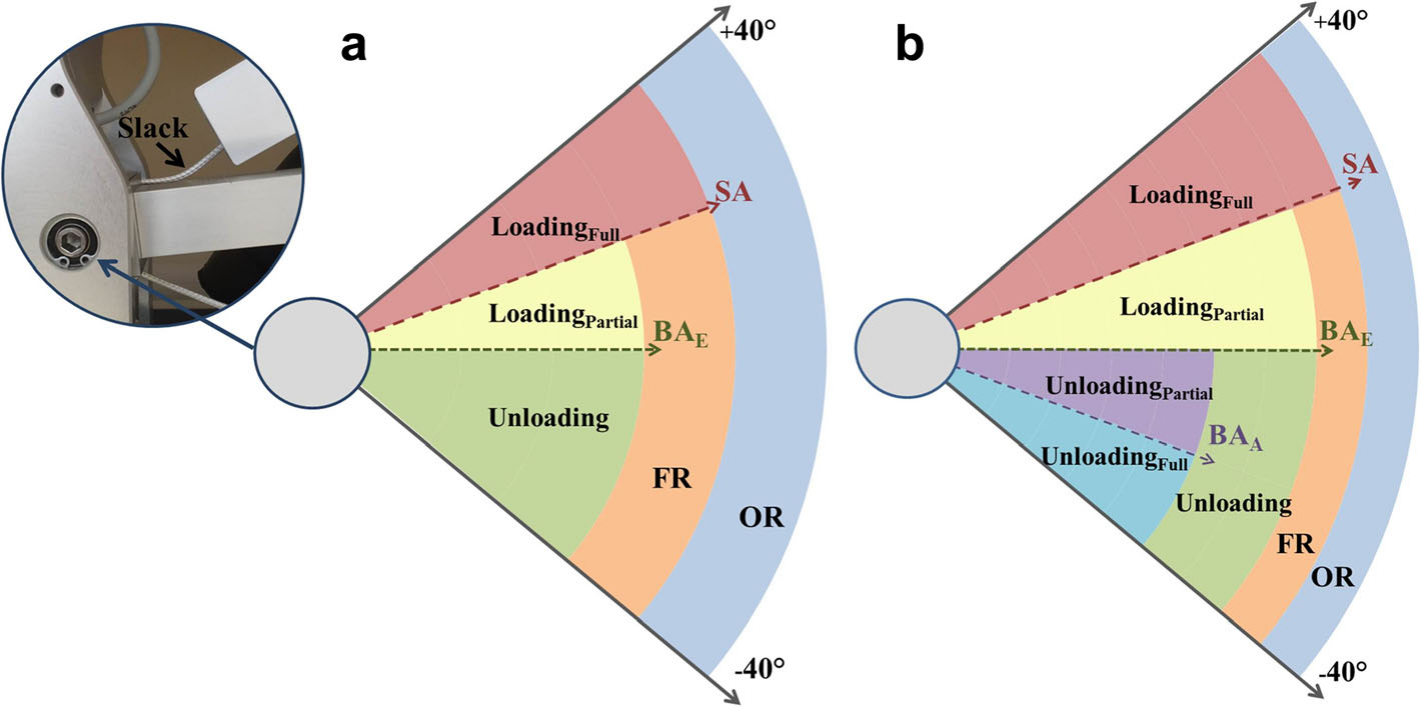
**a** A picture of the slack in the cable connecting the upper spring to the upper module and schematic representation of weight compensation regions of the exoskeleton alone, as described in the Methods. **b** Weight compensation regions after fitting arm into the exoskeleton, as described in the Discussion (SA, slack angle; BA_E_, exoskeleton balance angle; BA_A_, arm balance angle; FR, functional range; OR, operating range)

Due to the specific design and depending on the settings, the cable attaching the spring to the upper module begins to lose tension, which eventually disengages the spring (Fig. 2a-insert). The operating angle at which this occurred was termed the slack angle. Measurements were also taken above the slack angle to document exoskeleton weight and the spring functional range.

### Data analysis

Voltages recorded over 10 s epochs were averaged and converted to weight (kg). For the unloading condition, the weight was increased by 0.36 kg to account for instrumentation. Weight data for different spring and length settings were plotted against the upper module angle (elevation angle) to construct the respective weight-angle curves. The slack angle was identified as the sharp downward inflection point on the curve. Each weight-angle curve was fitted with a linear function between−40° and the slack angle, if present, to derive the slope and the x-axis intercept. The Additional file 1: Table S1 contains weight compensation data for each examined upper and lower spring/length setting used for generating weight-angle curves. Outcome measures were derived as defined below.

### Outcome measures

**Fig. 3:**
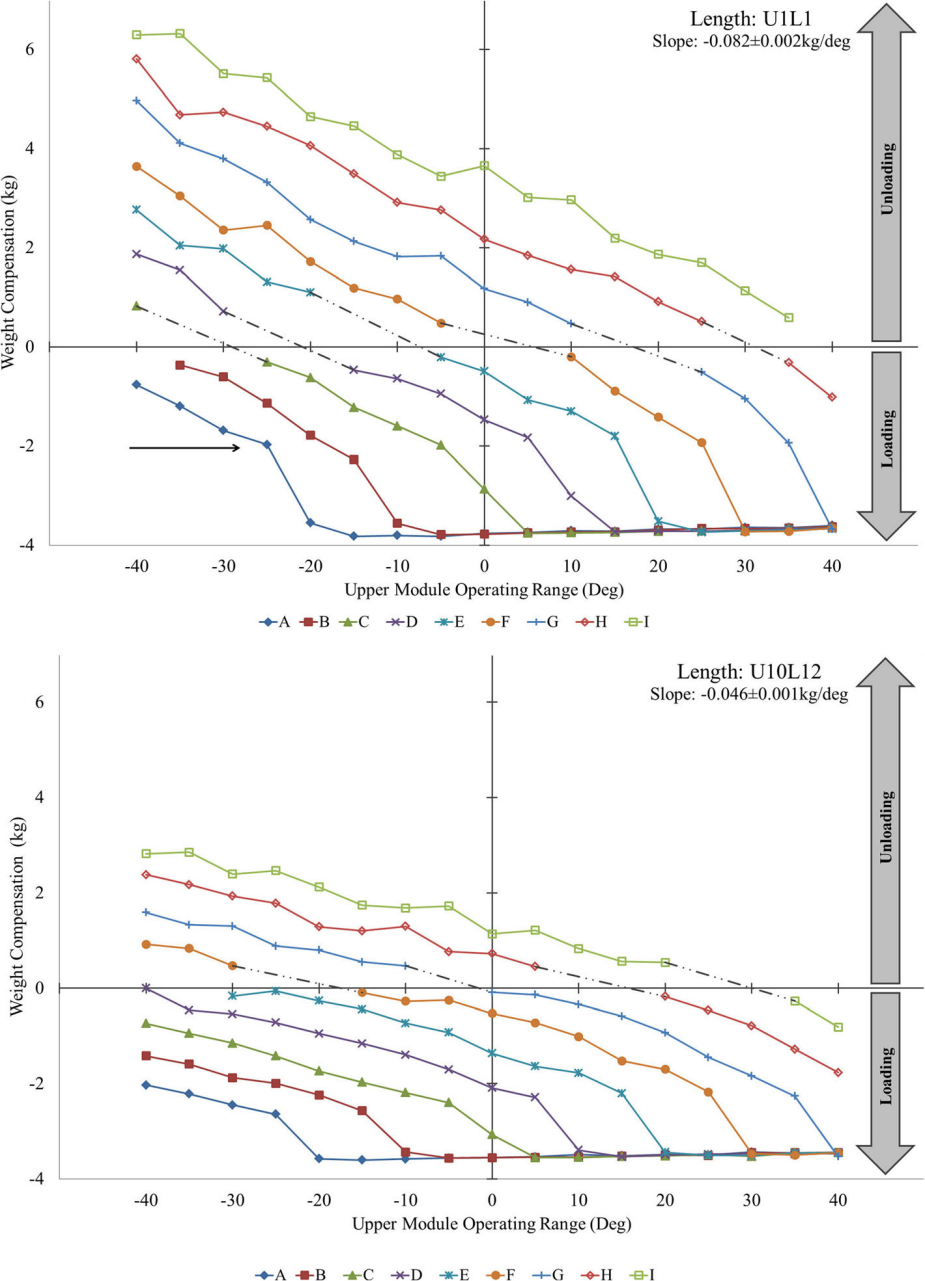
Weight compensation provided by upper module spring settings A-I across the operating range for upper (U) and lower (L) module lengths U1L1 (top) and U10L12 (bottom). Note the decrease in average slope and distribution of unloading (positive) and loading (negative) regions across different spring settings as a result of increasing module length. Additional file 1: Table S1 available online contains individual data for all examined spring and length settings and corresponding figure (Additional file 1: Figure S1) for module lengths U4L5 and U7L8

The outcome measures are schematically illustrated in Fig. 2a. The slack angle is the elevation angle at which the minimum weight compensation is provided before the upper spring becomes non-operational (expressed in 5° increments in the sagittal plane) (Fig. 3, top-arrow). The weight compensation (functional) range of the upper spring is the angular distance between the low end of the upper module operating range (−40°) and the slack angle. The exoskeleton balance angle is the elevation angle at which the full weight of the exoskeleton is supported by the spring (zero force exerted on the load cell), as determined by the x-intercept (Fig. 3). Unloading (positive weight compensation) is the amount of weight supported by the spring in excess of the weight of the exoskeleton. Loading (negative weight compensation) is the amount of exoskeleton weight imposed after exceeding the spring capacity of specific settings. The slope of the weight-angle curve is the average rate of change in weight compensation over the spring functional range. Outcome measures were reported as maximum values or ranges.

### Statistical analysis

The slopes of weight-angle curves for spring settings A-I were compared between the four length settings (U1L1, U4L5, U7L8, U10L12) using one-way ANOVA with spring setting as the repeated factor. If the main effect was significant (*p* < 0.05), Bonferroni’s adjustment was made for multiple comparisons. The summary results are reported as means ± standard errors.

## Results

### Slack angle

The slack angle was identified for upper spring settings A-G. It increased in 10° increments from−25° for setting A to +35° for setting G, regardless of module length settings (Table 1). The exceptions were−10° slack angle for spring setting C at length U4L5 and +30° for spring setting G at length U7L8. Slack angles for settings H and I could not be identified, as the cable remained taut throughout the entire operating range.

**Table 1:** Operating parameters of Armeo®Spring exoskeleton

	Upper Module Spring Settings								
	A	B	C	D	E	F	G	H	I
Slack Angle*	−25°	−15°	−5°	5°	15°	25°	35°	N/A	N/A
Functional Range*	15°	25°	35°	45°	55°	65°	75°	80°	80°
Full Loading Range*	65°	55°	45°	35°	25°	15°	5°	0°	0°
U1L1									
Exoskeleton Balance Angle	OOR	−37°	−29°	−18°	−7°	3°	15°	30°	OOR
Unloading Range	0°	3°	11°	22°	33°	43°	55°	70°	80°
Partial Loading Range	15°	22°	24°	23°	22°	22°	20°	10°	0°
U4L5									
Exoskeleton Balance Angle	OOR	OOR	−39°	−26°	−15°	−3°	10°	24°	OOR
Unloading Range	0°	0°	1°	14°	25°	37°	50°	64°	80°
Partial Loading Range	15°	25°	34°	31°	30°	28°	25°	16°	0°
U7L8									
Exoskeleton Balance Angle	OOR	OOR	OOR	−35°	−22°	−10°	−2°	18°	38°
Unloading Range	0°	0°	0°	5°	18°	30°	38°	58°	78°
Partial Loading Range	15°	25°	35°	40°	37°	35°	37°	22°	2°
U10L12									
Exoskeleton Balance Angle	OOR	OOR	OOR	OOR	−27°	−16°	−3°	12°	28°
Unloading Range	0°	0°	0°	0°	13°	24°	37°	52°	68°
Partial Loading Range	15°	25°	35°	45°	42°	41°	38°	28°	12°

*Applicable to all length configurations

Slack angle, functional range, and full loading range are consistent for all length configurations (U1L1, U4L5, U7L8, U10L12). Exoskeleton balance angle, unloading range, and partial loading range vary by spring (A-I) and length settings (OOR, outside operating range; N/A, not applicable)

### Spring functional range

Due to consistent slack angles for spring settings A-G, the functional range of the upper spring was also consistent regardless of length settings, from 15° to 75° (Table 1). Due to the absence of slack, the functional range for settings H and I coincided with the full operating range (80°).

### Balance angle of exoskeleton

Exoskeleton balance angles (Table 1) could not be determined because they were below the operating range for the length and spring settings U1L1 (A), U4L5 (A, B), U7L8 (A, B, C) and U10L12 (A, B, C, D), and above the operating range for U1L1 (I). Exoskeleton balance angles were within the operating range for spring settings E to H, regardless of length settings. The average increase in exoskeleton balance angle across all length settings was 14 ± 1° per increment in spring settings.

### Unloading

The unloading region is between the lower limit of the operating range (−40°) and the exoskeleton balance angle (Fig. 2a). Unloading (Fig. 3, above x-axis) was not provided by spring settings A for U1L1, A-B for U4L5, A-C for U7L8, and A-D for U10L12. When present, the maximum unloading was 6.32 kg for U1L1, 5.07 kg for U4L5, 3.53 kg for U7L8, and 2.85 kg for U10L12, all found at either−35° (I) or−40° (H) (Additional file 1: Table S1).

### Loading

The loading region is demarcated by the exoskeleton balance angle on the low end of the operating range and the slack angle on the high end (Fig. 2a). In the absence of slack angle, the loading region extends to the upper end of the upper module operating range (+40°). All spring and length settings imposed loading (Fig. 3, below x-axis), except spring setting I for U1L1 and U4L5. When averaged across all spring settings, the maximum loading (negative weight compensation) at the slack angle was−1.97 ± 0.06 kg (U1L1),−2.13 ± 0.08 kg (U4L5),−2.30 ± 0.07 kg (U7L8), and−2.36 ± 0.07 kg (U10L12). Immediately above the slack angle, the entire weight of the exoskeleton (3.59 ± 0.01 kg) is instantly imposed for the remainder of the operating range (denoted full loading region in Fig. 2a).

### Slope of weight-angle curve

All weight-angle curves (Fig. 3) were well fitted with the linear function (R^2^ = 0.96–0.99). The slopes for spring settings A-I significantly differed between the four length settings (p < 0.001), due to a decrease from −0.082 ± 0.002 kg/° (U1L1) to−0.066 ± 0.002 kg/° (U4L5),−0.052 ± 0.001 kg/° (U7L8), and−0.046 ± 0.001 kg/° (U10L12). All pairwise comparisons were also significant (p < 0.001), except between U7L8 and U10L12.

### Effect of lower spring settings

Changing the lower spring setting from A to E (while keeping the upper spring at A) had no effect on the slack angle and functional range of the upper spring. The exoskeleton balance angle measured−40° for U1L1 and remained outside the operating range for U10L12. As a result, no unloading was provided by either U1L1 or U10L12. The loading at the slack angle decreased from−1.98 kg (A) to−1.10 kg (E) for U1L1, but was comparable for U10L12 (about−2.70 kg each). Adjusting the lower spring from A to E did not affect the slope of the upper module weight-angle curve (−0.077 ± 0.004 kg/° U1L1,−0.040 ± 0.001 kg/° U10L12) (Additional file 1: ﻿Figure S2).

## Discussion

The results of this study confirm our hypothesis that weight compensation provided by the Armeo®Spring is mainly a function of spring tension and, to a lesser degree, module length settings. That is, higher spring tensions and shorter module lengths offer greater weight compensation.

The rate of change in weight compensation over the spring functional range was confirmed to be linear and consistent across settings A-I for each examined length setting. However, the rate of change was greater for shorter length settings (Fig. 3 top vs. bottom). For example, a 10° change in elevation angle results in 0.82 kg of unloading or loading using module length U1L1 but only 0.46 kg using U10L12. This occurs because the magnitude of weight compensation is determined by the difference in torque produced by the upward pull of the spring and the downward pull of the exoskeleton. For a given elevation angle, the downward pull of the exoskeleton increases with increasing module length (moment arm) resulting in less weight compensation. Thus, the change in weight compensation will differ along the range of elevation angles between persons who require different module lengths.

This study also revealed two other parameters relevant for appreciating the weight compensation characteristics of the Armeo®Spring, namely, the slack angle and the exoskeleton balance angle. Slack was observed at lower elevation angles with lower spring settings. It fell within the operating range for spring settings A-G but not H and I, regardless of module length (Table 1). This implies that when using spring settings A-G, the entire weight of the exoskeleton is instantly imposed beyond the slack angle.

The second relevant parameter is the exoskeleton balance angle. Similar to the slack angle, it mainly depends on the spring settings (the lower the tension, the lower the exoskeleton balance angle) and less on the module length settings. The practical significance of the exoskeleton balance angle is that it divides the spring functional range into two distinct regions (Fig. 2); the unloading region and the loading region. This means that the weight of the arm is supported in the unloading region, and the weight of the exoskeleton is imposed on the arm in the loading region. The magnitude of unloading progressively decreases from the lower end of the spring functional range toward the exoskeleton balance angle, whereas the magnitude of ploading progressively increases above the exoskeleton balance angle toward the upper end of the operating range (Fig. 3).

### Clinical implications

**Fig. 4:**
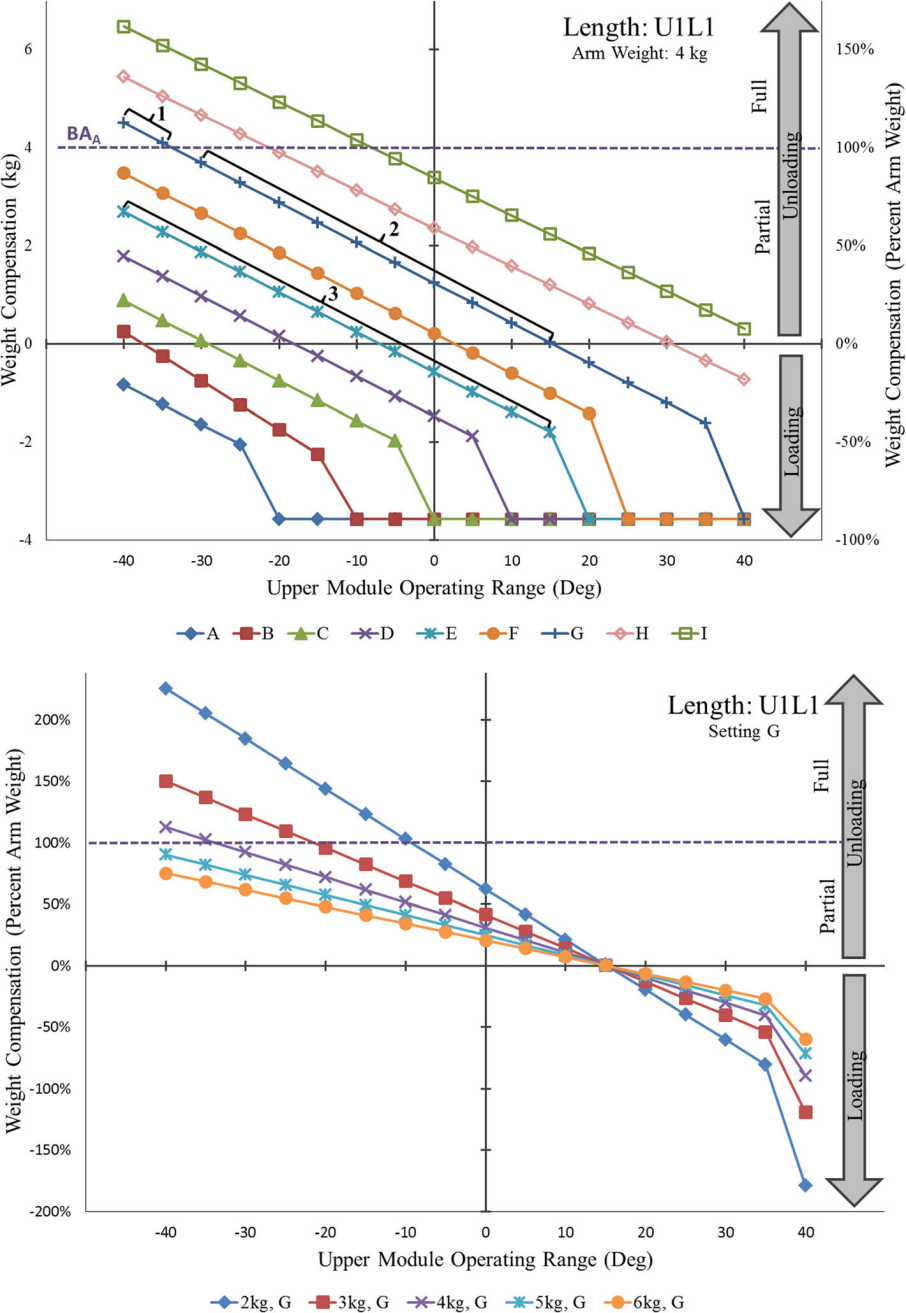
(Top) Absolute and percent weight compensation provided to a hypothetical 4 kg arm by upper module spring settings A-I for upper (U) and lower (L) module length U1L1. Bracket 1 marks the full unloading range for spring G. Bracket 2 marks the partial unloading range for spring G. Bracket 3 marks the combined unloading and loading range for spring E. (Bottom) Percent weight compensation provided to arms of three different weights by upper module spring G for upper (U) and lower (L) module length U1L1

Knowledge of the Armeo®Spring operating principles is a prerequisite for understanding the weight compensation provided to an arm once fitted into the exoskeleton. Adding arm weight introduces an additional operating parameter, the *arm balance angle*. It is the angle at which the combined weight of the exoskeleton and arm is fully supported by the spring. The practical relevance of the arm balance angle is twofold. First, it allows for estimating arm weight, which can be determined from the weight-angle curve using spring settings C and above (settings that balance the arm). Arm weight is equal to the amount of unloading that corresponds to the arm balance angle along the weight-angle curve of the selected spring. The arm balance angle can be observed and read from the software as the angle at which the exoskeleton comes to rest after fitting the arm into it. For example, if the arm balance angle is around−35° using spring setting G (U1L1), the weight of the arm would be approximately 4 kg (Fig. 4, top, an intersection of virtual line projected from−35° upward onto G weight-angle curve). Secondly, the arm balance angle divides the unloading region into *partial and full unloading regions* (Fig. 2b). For example, spring setting G (U1L1) provides full unloading from−35° to−40° for an arm weighing 4 kg because it can support between 4 and 5 kg within this range (Fig. 4, top-Bracket 1). The same spring setting G offers partial unloading between−30° and 15° since it can only support about 3.8 kg at−30° and gradually less thereafter (Fig. 4, top-Bracket 2). For the known arm weight, the arm balance angle can be determined for each spring as the intersection between the horizontal line designating arm weight and each weight-angle curve, estimated between−30° and−35° for spring setting G in this case (Fig. 4, top). This can help therapists plan and more specifically select different regions for exercise, depending on therapy goals.

**Fig. 5:**
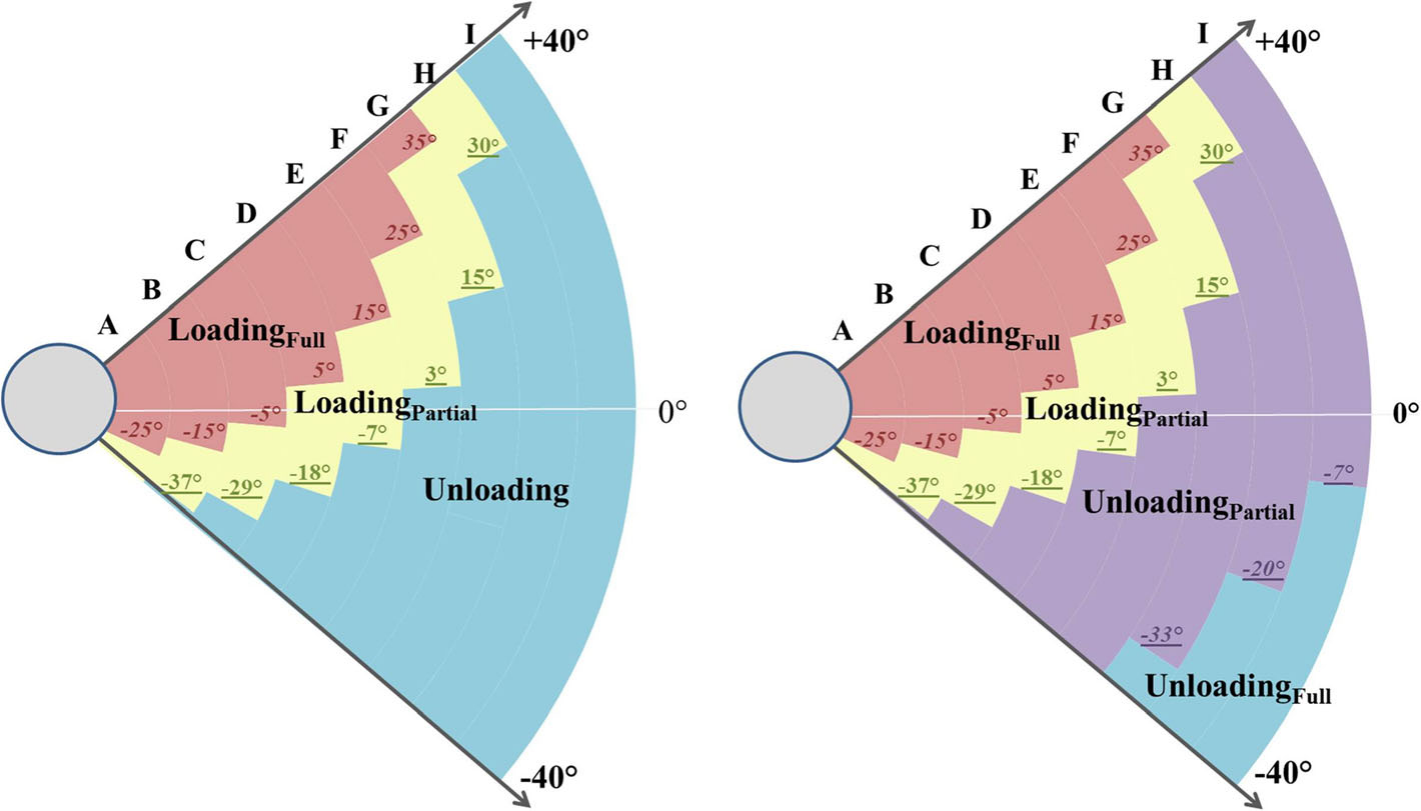
Schematic representation of the slack angles (italic), exoskeleton balance angles (underline), and arm balance angles (italic, underline) along with the resultant weight compensation regions (full loading, partial loading, full unloading, and partial unloading) across all spring settings A-I for upper (U) and lower (L) module length U1L1 for the exoskeleton without a user arm (left) and with a 4 kg arm (right)

The weight-angle curves are also useful to translate weight compensation from kilograms into percentages of arm weight and thereby arrive to the relative level of weight compensation. Expressing relative weight compensation allows for standardization of research protocols across participants and aids in the formulation of common therapy plans. Using spring setting G (U1L1), for example, the same arm weighing 4 kg can receive between 115% and 0% unloading from−40° to 15° (Fig. 4, top-Brackets 1 and 2). Over the same range, however, spring setting E (U1L1) provides between 70% unloading and 45% loading (Fig. 4, top-Bracket 3). Thus, spring settings should be selected depending on the relative degree of unloading or loading desired during exercise. Based on our results, the regions of unloading and loading can be defined for an individual user according to desired therapeutic goals (Fig. 5).

Further clinical implication of our results is that they can help to appreciate the relative support provided to arms of different weights, depending on the selected spring settings. Spring setting G (U1L1) provides over 100% unloading to arms weighing up to 4 kg (Fig. 4, bottom-above dash line), but only 75% unloading for a 6 kg arm (Fig. 4, bottom, below dash line). Thus, lighter arms receive relatively more support than heavier arms in the unloading region, but bear relatively more exoskeleton weight in the loading region. Moreover, the relative amount of loading and unloading changes faster for lighter than heavier arms within the same operating range. For example, a change in elevation of 10° will result in a change of approximately 40% in relative weight compensation for an arm weighing 2 kg but only 14% in relative weight compensation for an arm weighing 6 kg (Fig. 4, bottom). These arm weights correspond to body mass ranging from approximately 40 kg to 130 kg (for conversion, see Table 4 in [18]). Thus, spring settings need to be properly adjusted to achieve comparable levels of weight compensation for arms of different weight.

**Fig. 6:**
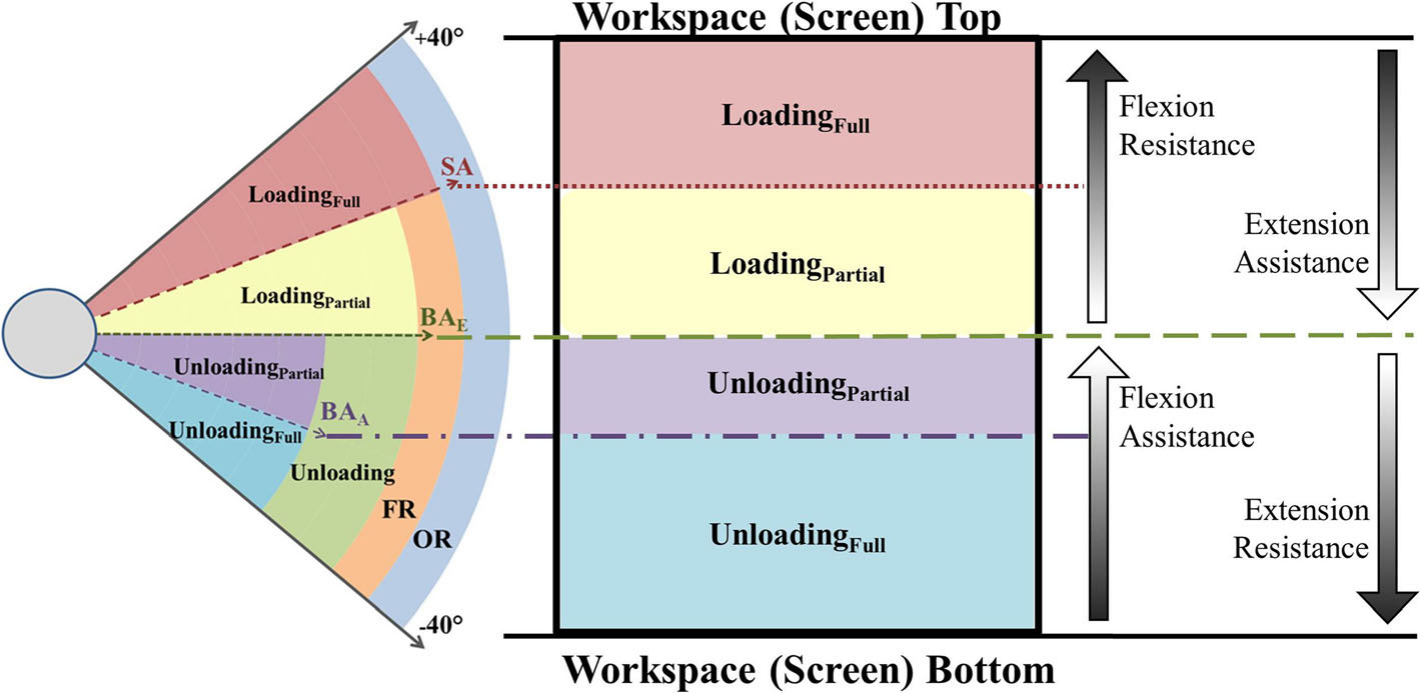
Schematic representation of the relationship between the regions of weight compensation and the virtual workspace projected on the computer screen, as described in the Discussion (SA, slack angle; BA_E_, exoskeleton balance angle; BA_A_, arm balance angle; FR, functional range; OR, operating range)

The operating parameters of the Armeo®Spring have, thus far, been discussed in the context of mechanical properties and their effects on the regions of loading and unloading. For practical purposes, this knowledge needs to be translated into the virtual workspace, which defines the exercise boundaries displayed on the computer screen. For example, setting the top and bottom workspace boundaries to correspond to the upper and lower limits of the operating range will permit exercises across all possible loading and unloading regions for the specified spring setting (Fig. 6). The location and size of different regions of loading/unloading on the screen will be delineated, starting from the bottom, by the lower limit of the operating range, arm balance angle, exoskeleton balance angle, slack angle, and upper limit of the operating range (Fig. 6). Within the entire workspace (screen), therefore, flexion and extension movements will be either assisted or resisted depending on the direction of movement and degree of loading/unloading provided by the selected spring setting to the arm of a given weight. Accordingly, the operating principles and settings of the Armeo®Spring should be carefully taken into account when configuring the virtual workspace in order to select exercise type and difficulty level commensurate with the residual muscle strength of upper arm flexors and extensors.

### Research implications

The Armeo®Spring intervention is administered in the context of user- and training-specific settings, such as arm size and weight, exoskeleton module length, spring tension, workspace, task selection, and level of difficulty, making it a rather complex intervention. To better appreciate its content, efficacy, and factors responsible for different outcomes, researchers may utilize the reported weight compensation properties of the Armeo®Spring in several ways. Our results may guide development of study aims that would attempt to isolate active ingredients of the treatment provided and draw a causal link with the outcome. With respect to study design and execution, the results provide a framework for predefining, standardizing, and consistently recording various relevant input parameters described here, as well as, for planning progress over time beyond dose, intensity, and frequency. In this way, better fidelity of intervention can be achieved, thus, strengthening validity of results. This would allow for more precise reporting of details of intervention applied, which has been a common problem in the field [19, 20], yet it is mandatory per the Consolidated Standards of Reporting Trials (CONSORT) guidelines [21]. Finally, this would make it easier to replicate studies, define next research steps, and conduct systematic analysis of Armeo®Spring outcomes.

### Study limitations

The reported results largely pertain to the upper spring and module settings, which we focused on due to the specific weight compensation design and greater need to compensate the weight of the upper arm than forearm. The same findings held in principle for the lower spring and module settings; however, their overall impact was much smaller. Only four module length settings were examined, but this was done in a systematic manner to anticipate the impact of other length settings. The impact of arm weight was not measured directly; however, the principles presented here were derived based on the laws of physics and can be observed while operating the device.

## Conclusions

This study confirmed that the weight compensation characteristics of the Armeo®Spring exoskeleton are primarily affected by spring tension and, to a lesser degree, module length settings. The design of Armeo®Spring prompted defining the slack and balance angles, which in turn demarcated regions of full and partial unloading and loading. These findings can be utilized in clinical practice for adjusting the Armeo®Spring settings to accommodate individual abilities and develop exercise programs according to therapy goals. In research, greater attention to and more precise selection of weight compensation as the primary input parameter of Armeo®Spring allows for better standardization of protocols, proper interpretation of outcomes, and compliance with the reporting guidelines.

## Additional file

Additional file 1: Table S1. Weight compensation (kg) provided by the upper module spring settings A-I for 4 upper (U) and lower (L) module lenghts of Armeo^®^Spring with the lower module spring at A. Last row of the tables U1L1 and U10L12 shows in addition weight compensation when the lower module spring was changed to E and the upper module spring at A. **﻿Figure S1.** Weight compensation (kg) provided by upper module spring settings A-I for upper (U) and lower (L) module lengths U4L5 (top) and U7L8 (bottom). **Figure S2.** Weight compensation (kg) provided by upper module spring setting A with lower module spring setting A and E for upper (U) and lower (L) module lengths U1L1 (top) and U10L12 (bottom). (DOCX 395 kb)

## Abbreviations

U10L12: Upper module length 10, Lower module length 12
U1L1: Upper module length 1, Lower module length 1
U4L5: Upper module length 4, Lower module length 5
U7L8: Upper module length 7, Lower module length 8
