# Supplementary material for: Weight compensation characteristics of Armeo®Spring exoskeleton: implications for clinical practice and research
Source: J Neuroeng Rehabil. 2017 Feb 17;14:14. doi: 10.1186/s12984-017-0227-0 (PMC5316193; doi:10.1186/s12984-017-0227-0)

Table S1: Weight compensation (kg) provided by the upper module spring settings A-I for 4 upper (U) and lower (L) module lenghts of Armeo^®^Spring with the lower module spring at A. Last row of the tables U1L1 and U10L12 shows in addition weight compensation when the lower module spring was changed to E and the upper module spring at A.

|  | Upper Module Operating Range (Degrees) | | | | | | | | | | | | | | | | |
| --- | --- | --- | --- | --- | --- | --- | --- | --- | --- | --- | --- | --- | --- | --- | --- | --- | --- |
|  | **-40** | **-35** | **-30** | **-25** | **-20** | **-15** | **-10** | **-5** | **0** | **5** | **10** | **15** | **20** | **25** | **30** | **35** | **40** |
| U1L1 |  |  |  |  |  |  |  |  |  |  |  |  |  |  |  |  |  |
| A : A | -0.77 | -1.20 | -1.69 | -1.98 | -3.55 | -3.82 | -3.81 | -3.82 | -3.77 | -3.74 | -3.71 | -3.72 | -3.68 | -3.68 | -3.64 | -3.65 | -3.61 |
| B : A | -- | -0.37 | -0.61 | -1.14 | -1.79 | -2.28 | -3.56 | -3.79 | -3.78 | -3.75 | -3.73 | -3.73 | -3.70 | -3.67 | -3.66 | -3.66 | -3.63 |
| C : A | 0.82 | -- | -- | -0.31 | -0.62 | -1.22 | -1.60 | -1.99 | -2.87 | -3.76 | -3.75 | -3.75 | -3.72 | -3.72 | -3.71 | -3.67 | -3.65 |
| D : A | 1.87 | 1.54 | 0.72 | -- | -- | -0.47 | -0.65 | -0.95 | -1.47 | -1.84 | -3.01 | -3.73 | -3.72 | -3.72 | -3.70 | -3.67 | -3.65 |
| E : A | 2.76 | 2.04 | 1.97 | 1.31 | 1.10 | -- | -- | -0.21 | -0.49 | -1.08 | -1.30 | -1.80 | -3.52 | -3.74 | -3.72 | -3.69 | -3.67 |
| F : A | 3.64 | 3.04 | 2.35 | 2.45 | 1.72 | 1.19 | 0.97 | 0.48 | -- | -- | -0.20 | -0.89 | -1.42 | -1.94 | -3.73 | -3.72 | -3.66 |
| G : A | 4.97 | 4.11 | 3.79 | 3.32 | 2.57 | 2.12 | 1.82 | 1.83 | 1.17 | 0.90 | 0.47 | -- | -- | -0.51 | -1.05 | -1.95 | -3.68 |
| H : A | 5.81 | 4.68 | 4.73 | 4.45 | 4.06 | 3.49 | 2.91 | 2.76 | 2.17 | 1.84 | 1.56 | 1.41 | 0.91 | 0.51 | -- | -0.32 | -1.02 |
| I : A | 6.29 | 6.32 | 5.51 | 5.43 | 4.64 | 4.45 | 3.88 | 3.44 | 3.65 | 3.01 | 2.96 | 2.19 | 1.86 | 1.70 | 1.13 | 0.59 | -- |
| A : E | 0.05 | -- | -0.77 | -1.10 | -3.46 | -3.89 | -3.87 | -3.88 | -3.86 | -3.84 | -3.81 | -3.80 | -3.75 | -3.77 | -3.73 | -3.78 | -3.68 |
| U4L5 |  |  |  |  |  |  |  |  |  |  |  |  |  |  |  |  |  |
| A : A | -1.45 | -1.91 | -2.24 | -2.48 | -3.67 | -3.76 | -3.73 | -3.72 | -3.70 | -3.70 | -3.66 | -3.68 | -3.66 | -3.66 | -3.63 | -3.63 | -3.64 |
| B : A | -0.37 | -0.98 | -1.56 | -1.73 | -2.15 | -2.41 | -3.38 | -3.73 | -3.73 | -3.70 | -3.70 | -3.71 | -3.65 | -3.66 | -3.63 | -3.63 | -3.61 |
| C : A | -- | -0.24 | -0.67 | -0.92 | -1.22 | -1.66 | -1.95 | -3.23 | -3.71 | -3.72 | -3.70 | -3.68 | -3.68 | -3.66 | -3.65 | -3.63 | -3.60 |
| D : A | 1.11 | -- | -- | -0.17 | -0.48 | -0.85 | -0.98 | -1.33 | -1.78 | -2.10 | -3.44 | -3.68 | -3.69 | -3.68 | -3.65 | -3.66 | -3.62 |
| E : A | 1.76 | 0.95 | 0.84 | 0.47 | -- | -- | -0.18 | -0.45 | -0.89 | -1.34 | -1.58 | -2.00 | -3.54 | -3.66 | -3.67 | -3.67 | -3.64 |
| F : A | 2.41 | 1.80 | 1.31 | 1.33 | 0.92 | 0.70 | 0.64 | -- | -- | -0.18 | -0.59 | -1.11 | -1.60 | -2.00 | -3.66 | -3.65 | -3.65 |
| G : A | 3.12 | 2.99 | 2.34 | 1.94 | 1.75 | 1.55 | 1.27 | 1.17 | 0.65 | 0.50 | -- | -- | -0.36 | -0.91 | -1.31 | -1.97 | -3.64 |
| H : A | 5.07 | 3.78 | 3.37 | 3.18 | 2.84 | 2.32 | 2.17 | 1.84 | 1.61 | 1.31 | 1.10 | 1.00 | 0.45 | -- | -0.24 | -0.75 | -1.33 |
| I : A | 5.00 | 4.75 | 4.05 | 3.87 | 3.63 | 3.16 | 2.89 | 2.66 | 2.59 | 2.37 | 2.16 | 1.71 | 1.43 | 1.14 | 0.92 | -- | -0.07 |
| U7L8 |  |  |  |  |  |  |  |  |  |  |  |  |  |  |  |  |  |
| A : A | -1.88 | -2.16 | -2.38 | -2.61 | -3.65 | -3.66 | -3.64 | -3.61 | -3.61 | -3.58 | -3.56 | -3.57 | -3.56 | -3.54 | -3.54 | -3.49 | -3.50 |
| B : A | -1.22 | -1.44 | -1.62 | -1.88 | -2.12 | -2.50 | -3.45 | -3.65 | -3.63 | -3.60 | -3.60 | -3.56 | -3.56 | -3.55 | -3.53 | -3.51 | -3.51 |
| C : A | -0.49 | -0.74 | -0.94 | -1.24 | -1.53 | -1.78 | -2.02 | -2.34 | -3.38 | -3.61 | -3.61 | -3.58 | -3.56 | -3.55 | -3.54 | -3.50 | -3.50 |
| D : A | -- | -0.09 | -0.29 | -0.59 | -0.72 | -0.95 | -1.22 | -1.53 | -1.99 | -2.16 | -3.34 | -3.58 | -3.56 | -3.55 | -3.54 | -3.52 | -3.49 |
| E : A | 0.84 | 0.42 | -- | -- | -0.03 | -0.16 | -0.49 | -0.71 | -1.14 | -1.39 | -1.89 | -2.06 | -3.39 | -3.55 | -3.54 | -3.53 | -3.50 |
| F : A | 1.41 | 1.42 | 0.84 | 0.89 | 0.40 | 0.26 | -- | -0.08 | -0.35 | -0.56 | -0.94 | -1.39 | -1.72 | -2.23 | -3.55 | -3.54 | -3.56 |
| G : A | 2.20 | 1.77 | 1.82 | 1.66 | 1.39 | 0.92 | 0.75 | 0.63 | -- | -- | -0.29 | -0.67 | -1.26 | -1.60 | -2.19 | -3.54 | -- |
| H : A | 3.02 | 2.74 | 2.56 | 2.40 | 1.86 | 1.69 | 1.72 | 1.20 | 1.20 | 0.81 | 0.59 | -- | -- | -0.13 | -0.57 | -1.02 | -1.56 |
| I : A | 3.48 | 3.53 | 3.26 | 3.05 | 2.92 | 2.40 | 2.21 | 2.06 | 1.74 | 1.52 | 1.53 | 1.31 | 1.06 | 0.68 | -- | -0.05 | -0.37 |
| U10L12 |  |  |  |  |  |  |  |  |  |  |  |  |  |  |  |  |  |
| A : A | -2.03 | -2.22 | -2.45 | -2.64 | -3.58 | -3.61 | -3.58 | -3.56 | -3.56 | -3.53 | -3.49 | -3.51 | -3.49 | -3.50 | -3.44 | -3.46 | -3.47 |
| B : A | -1.42 | -1.59 | -1.88 | -1.99 | -2.24 | -2.57 | -3.43 | -3.56 | -3.56 | -3.55 | -3.54 | -3.52 | -3.50 | -3.51 | -3.45 | -3.45 | -3.45 |
| C : A | -0.73 | -0.95 | -1.15 | -1.42 | -1.74 | -1.97 | -2.19 | -2.40 | -3.07 | -3.56 | -3.55 | -3.53 | -3.52 | -3.49 | -3.53 | -3.45 | -3.45 |
| D : A | -- | -0.46 | -0.54 | -0.72 | -0.95 | -1.16 | -1.40 | -1.70 | -2.09 | -2.29 | -3.40 | -3.53 | -3.50 | -3.48 | -3.47 | -3.49 | -3.45 |
| E : A | -- | -- | -0.16 | -0.06 | -0.27 | -0.44 | -0.73 | -0.93 | -1.36 | -1.63 | -1.78 | -2.20 | -3.44 | -3.51 | -3.51 | -3.45 | -3.45 |
| F : A | 0.92 | 0.84 | 0.47 | -- | -- | -0.09 | -0.27 | -0.25 | -0.53 | -0.72 | -1.01 | -1.52 | -1.70 | -2.17 | -3.47 | -3.50 | -3.45 |
| G : A | 1.59 | 1.33 | 1.30 | 0.89 | 0.80 | 0.55 | 0.47 | -- | -0.09 | -0.14 | -0.34 | -0.59 | -0.93 | -1.45 | -1.84 | -2.26 | -3.53 |
| H : A | 2.38 | 2.17 | 1.93 | 1.78 | 1.29 | 1.20 | 1.30 | 0.77 | 0.72 | 0.45 | -- | -- | -0.17 | -0.46 | -0.78 | -1.27 | -1.77 |
| I : A | 2.82 | 2.85 | 2.40 | 2.47 | 2.12 | 1.74 | 1.68 | 1.72 | 1.14 | 1.22 | 0.83 | 0.56 | 0.54 | -- | -- | -0.27 | -0.81 |
| A : E | -2.13 | -2.26 | -2.45 | -2.73 | -3.63 | -3.59 | -3.57 | -3.57 | -3.55 | -3.53 | -3.53 | -3.51 | -3.49 | -3.50 | -3.49 | -3.46 | -3.44 |
| --Data not collected; *Weight Compensation at Slack Angle. | | | | | | | | | | | | | | | | | |

Figure S1: Weight compensation (kg) provided by upper module spring settings A-I for upper (U) and lower (L) module lengths U4L5 (top) and U7L8 (bottom).


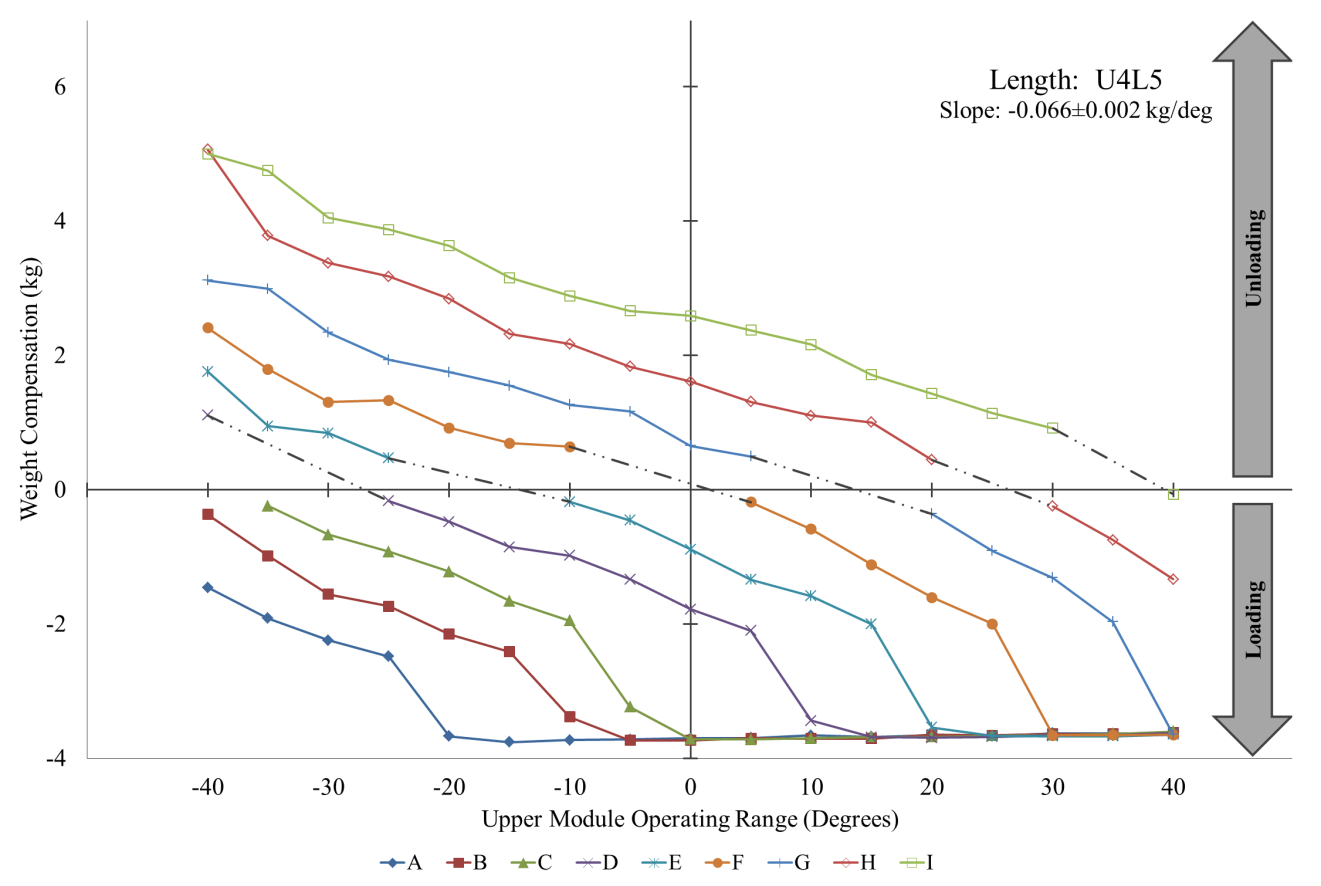


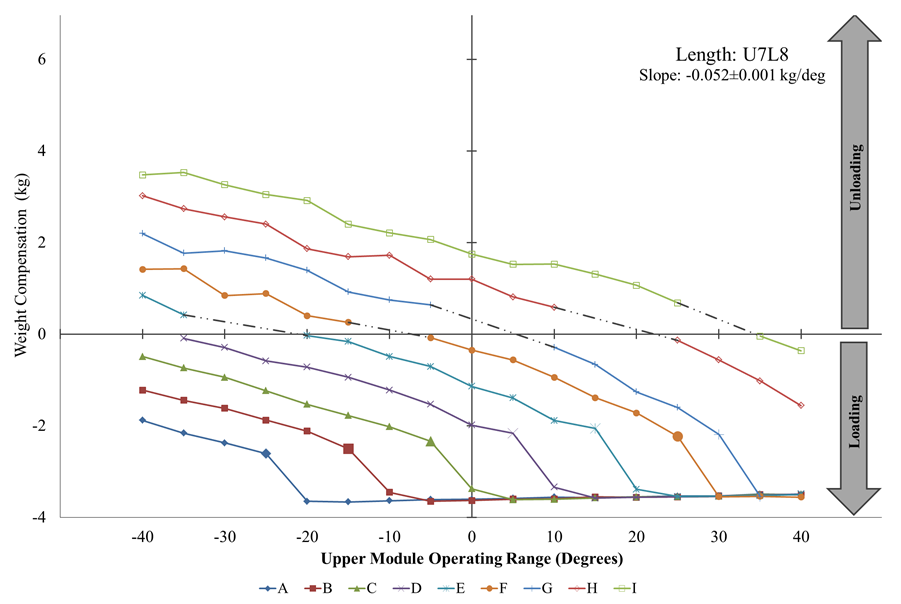


Figure S2: Weight compensation (kg) provided by upper module spring setting A with lower module spring setting A and E for upper (U) and lower (L) module lengths U1L1 (top) and U10L12 (bottom).


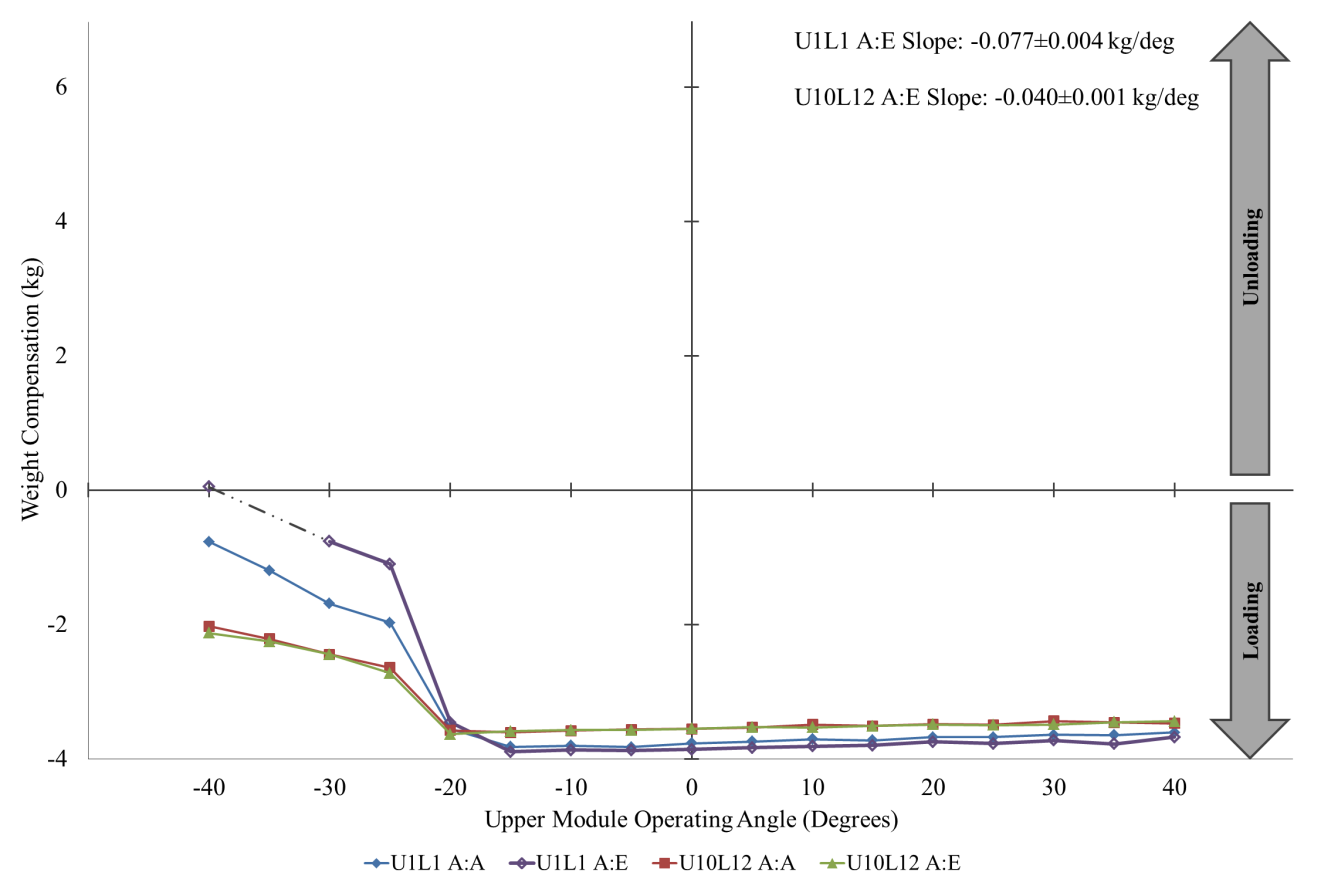

Supplement: Additional file 1: Table S1. — Weight compensation (kg) provided by the upper module spring settings A-I for 4 upper (U) and lower (L) module lenghts of Armeo®Spring with the lower module spring at A. Last row of the tables U1L1 and U10L12 shows in addition weight compensation when the lower module spring was changed to E and the upper module spring at A. ﻿Figure S1. Weight compensation (kg) provided by upper module spring settings A-I for upper (U) and lower (L) module lengths U4L5 (top) and U7L8 (bottom). Figure S2. Weight compensation (kg) provided by upper module spring setting A with lower module spring setting A and E for upper (U) and lower (L) module lengths U1L1 (top) and U10L12 (bottom). (DOCX 395 kb) [file 12984_2017_227_MOESM1_ESM.docx]
